# The Evolving Treatment Options for Diabetic Macular Edema

**DOI:** 10.1155/2013/689276

**Published:** 2013-09-09

**Authors:** Atul Jain, Neeta Varshney, Colin Smith

**Affiliations:** ^1^San Diego Retina Associates, 7695 Cardinal Court, Suite 100, San Diego, CA 92123, USA; ^2^Jules Stein Eye Institute, UCLA School of Medicine, Los Angeles, CA 90095, USA

## Abstract

Diabetic retinopathy (DR) is the leading cause of vision loss in working-age adults, and diabetic macular edema (DME) is the most common cause of visual impairment in individuals with DR. This review focuses on the pathophysiology, previous treatment paradigms, and emerging treatment options in the management of DME.

## 1. Introduction

Diabetic retinopathy (DR) is the leading cause of vision loss in working-age adults. In 2002, there were estimated to be just over 13.5 million individuals afflicted with diabetes mellitus (DM) in the USA, or about 6% of the population. Since then, revised estimates for 2011 indicate that 25.8 million people have DM in the USA, of which 18.8 million are diagnosed and 7 million cases are undiagnosed [1, 2]. Approximately 28.5% of individuals with DM have some form of retinopathy; 4.4% of individuals are at risk of severe vision loss secondary to advanced disease. Present estimates indicate that the incidences of DM and DR are both significantly increasing with as many as 50 million or more individuals in the USA having DM by the year 2050, of which half are expected to have some form of retinopathy [1–5]. 

DR can be categorized into two broad groups: (1) nonproliferative diabetic retinopathy (NPDR) and (2) proliferative diabetic retinopathy (PDR). Within NPDR, patients are classified as mild, moderate, or severe; severe NPDR is based on at least one of the following findings: diffuse intraretinal hemorrhages in all quadrants, venous beading in at least 2 quadrants, or the presence of intraretinal microvascular abnormalities. Of the two broad categories, proliferative disease, while it is less common, results in more severe vision loss. In nonproliferative disease, the most common cause of vision loss is due to diabetic macular edema (DME). At present, individuals with DR in the USA have a prevalence of DME between 3 and 5%, with this percentage increasing with age [6].

A recent meta-analysis of 35 population-based studies pooling data from the USA, Europe, Asia, and Australia found that in individuals with DM the prevalence of any type of DR is 35%, with DME present in 7.5% and PDR present in 7.2% of individuals. These prevalence rates were found to be significantly higher in individuals with type 1 DM compared to type 2 DM [7]. In the USA, over 90% of individuals with DM are type 2 diabetics [8].

Summarizing the above data as it applies to the USA, at present, approximately 1.1 million individuals are at serious risk of sight-threatening vision loss from DR. Of these “at risk” individuals, DME is the major etiology of visual impairment or loss with approximately 900,000 individuals with active DME in the USA. A decrease in visual acuity (VA) is commonly used to assess the severity of DME. Fluorescein angiography (FA) has been used extensively to image and assess diabetic eye disease and is useful in the identification of specific areas to treat when using targeted macular laser photocoagulation. More recently, optical coherence tomography (OCT) has become the gold standard used to objectively assess and quantify DME; central macular thickness (CMT) is the most common OCT measurement used for comparative purposes in recent clinical trials. VA outcomes are the focus of this paper.

## 2. Inflammation and DME

DME is due to extracellular swelling typically in Henle's layer of the macula caused by breakdown of the blood-retinal barriers [3]. Previously, DME was defined as clinically significant macular edema (CSME) or not, and focal laser treatment was initiated only for CSME (defined as thickening of the retina at or within 500 microns of the center of the macula, hard exudates at or within 500 microns of the center of the macula, if associated with thickening of adjacent retina, or a zone or zones of retinal thickening 1 disc area or larger of which any part is within 1 disc diameter of the center of the macula) [9]. More recently, DME has been subcategorized into two main categories: (1) focal diabetic macular edema (fDME) and (2) diffuse diabetic macular edema (dDME). With advancements in retinal imaging and an increased armamentarium of treatment options, the terms fDME and dDME may be more clinically relevant. Center-involving diabetic macular edema (ciDME) is also now commonly used to describe DME in which the central macula is involved.

As our knowledge of DME has advanced, we now know that the cause is multifactorial. Blood vessel damage plays a significant role in diabetics, both systemically and as related to the development of DME. Long-term hyperglycemia leads to vascular basement membrane thickening, nonenzymatic glycosylation, free radical formation, and pericyte death. These changes ultimately compromise the retinal vascular autoregulatory functioning leading to vascular dilation, increased capillary hydrostatic pressure, and microaneurysm formation [10]. The already weakened capillaries are further compromised due to the inflammatory changes known to occur in diabetics. The retinal vasculature of individuals with DM contains an increased density of leukocytes, which coincides with an increase in expression of ICAM-1 (intercellular adhesion molecule 1), also known as CD54 (cluster of differentiation 54) [11]. ICAM-1 can be induced by interleukin-1 (IL-1) and tumor necrosis factor alpha (TNF-*α*). ICAM-1 activation leads to proinflammatory changes and increased vascular permeability due to damage of vascular endothelial cells via a FasL-mediated mechanism leading to further breakdown of the blood-retinal barrier [12]. Numerous cytokines and proinflammatory factors have also been implicated as having a role in DME, the most studied of which is vascular endothelial growth factor (VEGF) [13, 14].  Table 1 lists the inflammatory factors which have been suggested to play a role in DME [15–23].

It is now well known that breakdown of the blood-retinal barrier results from compromised endothelial cell integrity. Osmotic fluctuations, due to hypertension and varying glycemic levels, increased vascular permeability, and capillary dropout, create an environment of inadequate blood flow to the retina. This retinal ischemia leads to the upregulation of VEGF, one of the most potent molecules in causing vascular permeability in humans [11]. VEGF mediates retinal vasculature hyperpermeability by opening endothelial tight junctions and inducing fenestrations. A compromised vascular endothelium secondary to ICAM-1 pathways in conjunction with damage caused by VEGF and other factors in the already weakened diabetic retinal vasculature precipitates a vicious cycle resulting in the inappropriate extravasation of intravascular contents. 

While there is significant upregulation of proinflammatory factors in individuals with DME, there is also downregulation of antiinflammatory factors, in particular pigment epithelium derived growth factor (PEDF). Vitreous levels of the following proinflammatory molecules: VEGF, ICAM-1, interleukin-6 (IL-6), and monocyte chemoattractant protein 1 (MCP-1) increase in individuals with DME, while vitreous levels of the antiinflammatory molecule PEDF may be significantly lower in diabetics with severe DME compared to those with only minimal or no DME [24]. Interleukin-8 (IL-8) levels are elevated in the aqueous of individuals with macular edema secondary to diabetes, but not retinovascular occlusive disease. Furthermore, IL-8 levels are not affected by the administration of intravitreal anti-VEGF or corticosteroid agents, indicating it could represent a new target in the management of DME [20]. 

## 3. Systemic Conditions and DME

Duration and control of DM play a major role in the development of DME. Individuals with a longer history of DM are at higher risk of developing DME as well as individuals with poor DM control (higher hemoglobin A_1_C concentrations) [3, 25]. Optimal hypertensive and DM control can delay and even prevent the onset of DME and vision loss. 

The Diabetes Control and Complications Trial (DCCT) evaluated patients with type 1 (insulin dependent) DM for 6.5 years and demonstrated that intensive glycemic control reduced the risk of developing retinopathy by 76% (10.7% versus 33.2%, intensive versus conventional control groups, resp.) in those with no previous retinopathy and slowed the progression of retinopathy by 54% in those who had mild DR. The conventional group had a hemoglobin A_1_C of 9.1 versus 7.2 in the intensive control group. At the closeout of the DCCT study, 3.9% (intensive group) versus 7.7% (conventional group) developed CSME [26–28]. The Epidemiology of Diabetes Interventions and Complications (EDIC) Research Group followed patients for 4 years after conclusion of the DCCT and found that the benefits of intensive diabetes control persisted even with increasing hyperglycemia (hemoglobin A_1_C increased to 7.9 in the intensive group, compared with a reduction to 8.2 in the conventional group). After four years of follow-up in the EDIC study, 18% of the patients in the intensive-therapy group had a progression in DR compared to 49% of the patients in the conventional-therapy group. At the closeout of the EDIC study, 3.8% (intensive group) versus 13.3% (conventional group) developed CSME [29]. At 10 years after the conclusion of the DCCT study, both intensive and conventional groups had a hemoglobin A_1_C of 8, with 36% of patients in the intensive group demonstrating a progression of DR compared to 61% in the conventional group. In the intensive group, 9% developed CSME and 8.9% developed PDR compared to 19% developing CSME and 24.7% developing PDR in the conventional group [30].

The United Kingdom Prospective Diabetes Study (UKPDS) studied the effects of glycemic control on type 2 (non-insulin dependent) diabetics and found that intensive glycemic control was associated with a 25% decrease in microvascular complications and a reduction in the need for macular laser photocoagulation. The UKPDS also found that intensive control of blood pressure (BP) had a 34% reduction in the risk of DR progression and a 37% reduction in diabetic microvascular endpoints, such as the need for retinal photocoagulation [31, 32].

## 4. Laser DME Treatment Paradigms

Until the early 1980s, there was no intervention available for the treatment of DME. A landmark prospective randomized study performed by the Early Treatment Diabetic Retinopathy Study (ETDRS) group found that grid macular photocoagulation decreased the risk of moderate to severe vision loss from DME by 50% compared to untreated controls over 3 years [33]. This was the standard of care for over 2 decades. Since the original ETDRS study, there has been evidence to support that a modified ETDRS laser technique has slightly better visual outcomes than a grid pattern of laser alone. In the modified technique, a light macular grid is performed in addition to the targeted treatment of microaneurysms with laser photocoagulation [34].

 There is some pieces of evidence that very short duration focal macular laser photocoagulation and subthreshold micropulse diode laser treatments are just as effective as the modified ETDRS method of laser treatment for DME, but with less collateral damage, a lower risk of inducing choroidal neovascularization, and less likelihood of laser wound creep into the central fovea [35–37].

The goal of focal macular laser photocoagulation is preservation of VA and prevention of severe VA loss (≥15 ETDRS letters, or 3 Snellen lines of VA) over the long term. Visual acuity gains from focal laser treatment are frequently modest with most studies reporting that 40% of eyes gain between 0 and 5 ETDRS letters over a two-year period [38–41].

## 5. Pharmacological DME Treatment Paradigms

 Corticosteroids were the first pharmacologic intravitreal treatment to be used for DME. Corticosteroids reduce vascular permeability of the retina; while their exact mechanism of action is not completely understood, they reduce production of arachidonic acid derivatives such as prostaglandins as well as inhibiting ICAM-1, TNF-*α*, and VEGF [3, 11, 37].

 Triamcinolone acetonide has been the most widely used and studied corticosteroid in the treatment of DME [39, 42–44]. More recently, other formulations of corticosteroids have been studied and found to be effective in the reduction of DME, including a biodegradable dexamethasone implant (Ozurdex; Allergan, Irvine, CA), a time-released nonbioerodible surgically implantable reservoir of fluocinolone (Retisert; Bausch & Lomb, Rochester, NY), and a non-bioerodible injectable fluocinolone polymer (Iluvien; Alimera Sciences, Alpharetta, GA) [45–49]. None of the corticosteroids mentioned are currently Food and Drug Administration (FDA) approved for the treatment of DME.  Table 2 lists the results of the major studies evaluating corticosteroids for the treatment of DME [39, 43, 46–48, 50].

 Intravitreal triamcinolone acetonide has been used for the treatment of DME for a number of years. The effects are often short-lived, requiring frequent retreatment with the main side effects being cataract and glaucoma. In eyes with DME, use of both 2 mg and 4 mg doses resulted in over 50% of eyes gaining ≥10 ETDRS letters (2 lines of Snellen VA), with the effects lasting for 16 and 20 weeks, respectively [42]. In 2-year follow-up of eyes with DME refractory to macular laser, eyes that received 4 mg of intravitreal triamcinolone acetonide gained 3.1 ETDRS letters compared to a loss of 2.9 ETDRS letters in the placebo group [43]. When comparing 2-year VA outcomes of focal macular laser alone to 1 mg versus 4 mg intravitreal injections of triamcinolone acetonide, it was found that laser was superior. Eyes treated with macular laser photocoagulation gained a mean of 2 ETDRS letters compared to a loss of 2 and 4 ETDRS letters in the 1 mg and 4 mg triamcinolone groups, respectively. At 3 years, the laser only group continued to fare better with a gain of 5 ETDRS letters compared to a 0 letter gain in both 1 and 4 mg triamcinolone groups [39, 44].

 A Phase 2 clinical trial evaluating the safety and efficacy of a 0.59 mg surgically implanted fluocinolone acetonide intravitreal implant (Retisert) in eyes with DME found that VA gains of ≥15 ETDRS letters occurred in 16.8% of implanted eyes at 6 months and 31.1% of eyes at 3 years, compared to 1.4% at 6 months and 20% at 3 years in the macular laser group. The results were significant at the 6 month time point (*P* = 0.002) but not at 3 years (*P* = 0.16). The incidence of elevated intraocular pressure and cataract formation was much higher in eyes receiving the implant with 33.8% requiring incisional glaucoma surgery and 91% requiring cataract extraction compared to 0% and 20% in the standard of care group (observation or laser), respectively. Retisert is FDA approved for use in chronic, noninfectious uveitis [46].

A Phase 3 clinical trial evaluating the efficacy and safety of an intravitreally injected fluocinolone acetonide insert (Iluvien) in eyes with DME at low (0.2 *μ*g/d) and high (0.5 *μ*g/d) doses found VA gains at 3-years of ≥15 ETDRS letters in 33% and 31.9% of study eyes, respectively, while 21% of eyes in the sham injection group had a ≥15 ETDRS letter gain at 3 years (*P* = 0.030). Of treated eyes, 26% required more than one treatment over the 3 year period. Cataract surgery was required in 83.8% of eyes in the treatment groups compared to 27.3% in the sham group. The incidence of elevated intraocular pressure was much higher in the treatment groups with 4.8% (low dose) and 8.1% (high dose) of eyes requiring incisional glaucoma surgery compared to 0.5% in the sham group [47, 48]. While the 0.2 *μ*g/d dose of Iluvien is approved for use in many European countries (Austria, the United Kingdom, Portugal, France, Germany and Spain), it has yet to be approved for use in the United States. 

A Phase 2 clinical trial evaluating the efficacy and safety of a surgically implanted intravitreal dexamethasone delivery system in eyes with DME found that a 700 *μ*g dose resulted in VA gains of ≥10 ETDRS letters at 90 days after implantation in 33.3% of eyes and 30% of eyes at 180 days. In the 350 *μ*g group, ≥10 ETDRS letter gains were seen in 21.1% and 19% at 90 and 180 days after implantation, respectively. In the control (observation) group, ≥10 ETDRS letter gains were seen in 12.3% and 23% of eyes at 90 and 180 days, respectively. The only statistically significant difference between treatment versus control groups at day 90 was in the 700 *μ*g treatment group (*P* = 0.007). There was no significant increase in cataract development between treatment and control groups. The treatment group did have a higher incidence of elevated intraocular pressure compared to the control group, but no incisional glaucoma surgery was required in any eyes study [49]. A Phase 3 study of an injectable form of this biodegradable implant (Ozurdex) is currently ongoing.

 VEGF-A is believed to be one of the major mediating factors associated with the development of DR and DME. VEGF is a proinflammatory mediator and plays a pivotal role in vascular permeability. It is well known that VEGF levels are higher in diabetic eyes than in normal eyes [51]. At present, there are 4 medications available that target VEGF-A: pegaptanib (Macugen; Eyetech Pharmaceuticals, Palm Beach Gardens, FL, USA), bevacizumab (Avastin, Genentech, San Francisco, CA, US), ranibizumab (Lucentis; Genentech, San Francisco, CA, US), and aflibercept (Eylea; Regeneron, Tarrytown, NY) [40, 52, 53].  Table 3 lists the results of the major studies evaluating anti-VEGF agents for the treatment of DME [40, 41, 53–59].

 Pegaptanib, a pegylated aptamer that targets the VEGF-165 isoform, when administered intravitreally every 6 weeks was found to be more efficacious than macular laser at 24 months, with ETDRS letter gains of 6.1 and 1.3, respectively [52]. Intravitreal bevacizumab, a full-length recombinant humanized antibody against all isoforms of VEGF-A, was found to be more effective than macular laser for persistent ciDME at 24 months, with ETDRS letter gains of 8.5 and −0.5, respectively [40]. Neither pegaptanib nor bevacizumab is approved by the FDA for the treatment of DME though bevacizumab is widely used for this indication. Pegaptanib is FDA approved for the treatment of neovascular age-related macular degeneration (AMD).

 In August 2012, ranibizumab, a recombinant humanized monoclonal antibody fragment that binds all isoforms of VEGF-A, was approved by the FDA for the treatment of DME at the 0.3 mg dose, administered monthly via intravitreal injection. Treatment with ranibizumab resulted in over 39% of eyes with visually significant DME gaining ≥15 ETDRS letters or more of vision compared to only 18% of control eyes (which were eligible for macular laser photocoagulation based on protocol specific criteria). The overall gain in VA with monthly ranibizumab injections was 10.9 and 12 ETDRS letters in the 0.3 mg and 0.5 mg groups, respectively, compared to a 2.3 letter gain in the control group. Individuals with a hemoglobin A_1_C level ≤8 had a higher likelihood of a ≥15 letter gain than individuals with higher hemoglobin A_1_C levels. Results were sustained for 24 months with continued treatment [53]. 

 The most recent anti-VEGF agent which has been introduced is aflibercept, previously known as the VEGF-Trap-Eye and is currently approved in the USA for the treatment of neovascular AMD and macular edema secondary to central retinal venous obstruction. Aflibercept binds both VEGF-A and placental growth factors 1 and 2, is delivered via intravitreal injection and is currently under study for the treatment of DME. Initial one year results demonstrate that over 40% of eyes with visually significant DME gained at least 3 lines of vision compared to 11.4% in the macular laser control group [58].

Given the results from studies with both corticosteroids and anti-VEGF agents, the goal in treatment of DME is now preservation and improvement in VA instead of just maintenance or reduction in the amount of vision loss as was the case with macular laser photocoagulation, the previous standard of care.

## 6. Combination Therapy for DME

Intravitreal pharmacotherapy has replaced macular laser photocoagulation as the gold standard in the care of DME. While it is quite successful in preventing vision loss from DME, and allowing for a significant number of people to realize a gain in VA, the burden of monthly intravitreal injections can become quite an encumbrance for patients, physicians, and the healthcare system as a whole due to high costs of medications, multiple physician visits, and potential complications from an invasive procedure. This has prompted studies to evaluate if combination therapies with both laser and intravitreal injections can be more efficacious than either treatment alone or if combination therapy allows for fewer treatments while maintaining VA gains. A large prospective, randomized, double-blinded study conducted by the Diabetic Retinopathy Clinical Research Network (DRCR) sought to answer this specific question. Eyes with DME were treated with focal macular laser photocoagulation alone, 0.5 mg of monthly ranibizumab + prompt focal macular laser, 0.5 mg of monthly ranibizumab + deferred focal macular laser (after week 24), or 4 mg of quarterly triamcinolone acetonide + prompt focal macular laser. After the first year, intravitreal medications were only administered as needed based on clinical examination. At the end of the 2-year study, it was found that ranibizumab + deferred focal macular laser was the superior treatment algorithm for eyes with visually significant DME. In the ranibizumab + deferred laser group 28% of eyes gained ≥15 ETDRS (mean gain = 9 letters); in the ranibizumab + prompt laser group 29% of eyes gained ≥15 ETDRS letters (mean gain = 8 letters); a median of 2 and 3 ranibizumab injections were required the second year for the deferred versus prompt groups, respectively. In the laser only group, 18% of eyes gained ≥15 ETDRS letters with a mean VA gain of 3 letters. In the triamcinolone + laser group, 22% of eyes gained ≥15 ETDRS letters, with a mean VA gain of 2 letters [60]. 

 A 2-year retrospective study evaluating bevacizumab versus bevacizumab + macular laser versus macular laser alone for eyes with DME found that the bevacizumab only group did better than the other groups with gains of 11.8 ETDRS letters compared to 8.2 and 4.8 ETDRS letter gains, respectively. There was no statistically significant difference between the bevacizumab and bevacizumab + macular laser group, but both these groups were statistically superior to the macular laser only group [61]. The retrospective nature of this study limits the conclusions that can be drawn, and the number of intravitreal treatments in the bevacizumab groups was not indicated.

 Anti-VEGF agents have changed how DME is managed providing patients with significant VA gains that are sustainable with repeat injections. Combination therapy is an evolving field and further research is needed to determine how best to care for patients with DME. Given the multifactorial nature of DME, additional studies are necessary to evaluate the role of combination therapy of anti-VEGF agents with corticosteroids in an effort to alleviate the treatment burden of monthly dosing and to assess the efficacy in those individuals with persistent DME despite repeated anti-VEGF therapy. Macular laser photocoagulation still has a role in DME, particularly fDME; however, the optimal timing of when to initiate treatment needs to be further elucidated. 

## 7. Other and Emerging Treatments for DME

 The vitreous humor has been implicated as a cause of DME due to an increase in the concentration of factors affecting vascular permeability as well as the exertion of tractional forces on the macula [62]. The role of pars plana vitrectomy has been evaluated in the management of DME with mixed results with slightly more eyes gaining ≥10 ETDRS letters than losing the same amount (38 and 22%, resp.). The best outcomes were seen in eyes in which starting VA was lower and had an epiretinal membrane present prior to surgery (which was removed at the time of vitrectomy) [63, 64]. 

Use of pharmacologic therapy after vitrectomy in patients with persistent DME remains challenging as clearance of drugs is more rapid in vitrectomized eyes. In a retrospective study of 11 vitrectomized eyes with DME, 3 monthly injections of bevaacizumb had no effect on mean VA or mean foveal thickness [65]. A single intravitreal injection of 0.7 mg dexamethasone (Ozurdex) in previously vitrectomized eyes with persistent DME demonstrated a VA gain of 6 ETDRS letters at week 8 and 3 ETDRS letters at week 26 [50]. In a small prospective study evaluating vitrectomy + intravitreal bevacizumab and triamcinolone acetonide versus vitrectomy + intravitreal bevacizumab and triamcinolone acetonide followed by focal macular laser 2 weeks later in eyes with intractable dDME, VA gains of approximately 10 ETDRS letters were realized in both groups 1 year after treatment [66].

 Due to the tractional component of the vitreous on the macula, induction of a posterior vitreous detachment (PVD) has shown some modest benefit in those with DME [67]. Ocriplasmin (Jetrea; ThromboGenics, Belgium) has been approved by the FDA for the treatment of vitreomacular adhesion and has some efficacy in inducing a PVD [67]. It is a serine protease which is injected into the vitreous and may have a beneficial role in the treatment for DME. Prospective studies to evaluate this are currently underway.

## 8. Conclusion

 There has been an incredible advancement in the treatment of DME over the past 2 decades with the treatment paradigm changing from observation and macular laser photocoagulation to intravitreal pharmacologic therapies of corticosteroids and anti-VEGF agents. Physician and patients are now pursuing gains in VA instead of maintenance or reduction in rate of visual loss from DME.

The future of DME has numerous treatment options available for physicians and patients to not only maintain vision but also improve and maintain sustained VA gains. The future is promising and will likely be comprised of a combination approach utilizing anti-VEGF agents, laser, and corticosteroids designed to address the multifactorial nature of the disease. Thanks to advances in our understanding and increased treatment options for DME, we are now able to better manage this condition for affected patients. While DME was often blinding in the past, we now are able to provide many of our patients with excellent and sustained vision, thereby allowing them to continue to be a part of the workforce. The future is promising, but it must be kept in mind that DM is a systemic disease and optimal glycemic and BP control are of paramount importance in both preventing and delaying the progression of both DR and DME. Communication and a team approach among primary care physicians, endocrinologists, and ophthalmologists will allow patients with DME to achieve and maintain long-term sustained VA gains.

## Figures and Tables

**Table 1:** Inflammatory factors suggested to play a role in DME.

Reference	Factor	Abbreviation	Clinical relevance
[15]	Angiopoietin-1 and 2	Ang1/Ang2	Angiogenesis and neovascularization
[16]	Erythropoietin	Epo	Stimulates retinal endothelial cell proliferation
[17]	Hepatocyte growth factor	HGF	Stimulate: proliferation, migration, and invasiveness of retinal endothelial cells
[18]	High-sensitivity C-reactive protein	hsCRP	Possibly related to CSME and hard exudation
[19]	Insulin-like growth factor-1	IGF-1	Angiogenesis
[18]	Intercellular adhesion molecule 1	ICAM-1	Possibly related to CSME and hard exudation
[20]	Interleukin 6	IL-6	Vascular permeability
[20]	Interleukin 8	IL-8	Mechanism unknown, upregulated in DME but not macular edema from vascular occlusive disease
[20]	Monocyte chemoattractant protein 1	MCP-1	Leukostasis leading to hypoxia
[21]	Pigment epithelium-derived factor	PEDF	Antiangiogenic and antiinflammatory
[22]	Protein kinase C	PKC	Increases vascular permeability and contractility
[19]	Stromal-derived factor 1	SDF-1	Angiogenesis
[23]	Thrombospondins 1 and 2	TSP-1 and 2	Anti-angiogenic; inhibit endothelial cell proliferation and apoptosis
[20]	Vascular endothelial growth factor	VEGF	Angiogenesis and vascular permeability

**Table 2:** Summary of major studies evaluating corticosteroids for DME.

Reference	Study name	Follow-up	Type of DME	Type of study	Study methodology	Number of treatments	Mean ETDRS letter gains	Number of eyes
[39]	DRCR protocol B: triamcinolone versus laser	36 months	CMT OCT ≥ 250 *μ*m ciDME	Prospective, multicenter	Laser alone	3.1	5	115
					1 mg triamcinolone	4.2 IVI	0	93
					4 mg triamcinolone	4.1 IVI	0	98

[43]	Triamcinolone versus placebo for refractory DME	24 months	ciDME after ≥ 1 previous laser treatment	Prospective, multicenter	Placebo (sham IVI)	N/A	−2.9	29
					4 mg Triamcinolone	2.6	3.1	31

[46]	Flucinolone acetonide	36 months	CSME after ≥ 1 previous laser	Prospective, multicenter, Phase 2	0.59 mg flucinolone acetonide surgical implant	1	31% ≥ 15 letter gain	127
	Intravitreal implant for DME (Retisert)				Standard of care (observation or laser)	Not stated	20% ≥ 15 letter gain	69
					Note: rescue macular laser for both groups			

[47]	**FAME ^*∧*^(Iluvien)	36 months	CMT OCT ≥ 250 *μ*m after ≥ 1 previous laser	Prospective, multicenter	0.5 *μ*g fluocinolone acetonide intravitreal insert	1.3 IVI; ≥3 laser in 3.3%	7.1	270
					0.2 *μ*g fluocinolone acetonide intravitreal insert	1.2 IVI; ≥3 laser in 6.6%	8.1	276
					Sham	≥3 laser in 11.9%	3.1	126
					Note: rescue macular laser after week 6			

[49]	***Dexamethasone Drug	6 months	CSME after ≥ 1 previous laser	Prospective, multicenter, Phase 2	700 *μ*g dexamethasone surgical implant	1	33.3% ≥ 10 letter gain^*∧*^^*∧*^	57
	Delivery system in DME (Ozurdex)				350 *μ*g dexamethasone surgical implant	1	21.1% ≥ 10 letter gain	57
					Observation	N/A	12.3% ≥ 10 letter gain	57

[50]	Dexamethasone drug Delivery system in vitrectomized patients	6 months	CMT OCT ≥ 275 *μ*m with history of vitrectomy	Prospective, multicenter, Phase 2	0.7 mg dexamethasone IVI	1	3	56

*IVI: intravitreal injection.

**Specific number of laser treatments not stated.

***Specific letter gains not stated.

^*∧*^Trade name of medication used is indicated in parentheses ().

^*∧*^^*∧*^Primary endpoint was day 90 and 10 letter gain.

**Table 3:** Summary of major studies evaluating anti-VEGF medications for DME.

Reference	Study name	Follow-up	Type of DME	Type of study	Study methodology	Number of treatments	Mean ETDRS letter gains	Number of eyes
[53]	RIDE	24 months	CMT OCT ≥ 275 *μ*m	Prospective, multicenter, Phase 3	Sham	1.6 laser	2.3	130
					0.3 mg lucentis	20.5 IVI; 0.7 laser	10.9	125
					0.5 mg lucentis	21.9 IVI; 0.3 laser	12	127
					Note: rescue laser after month 3			

[53]	RISE	24 months	CMT OCT ≥ 275 *μ*m	Prospective, multicenter, Phase 3	Sham	1.8 laser	2.6	127
					0.3 mg lucentis	21.5 IVI; 0.8 laser	12.5	125
					0.5 mg lucentis	20.9 IVI; 0.8 laser	11.9	125
					Note: rescue laser after month 3			

[54]	RESTORE	12 months	fDME and dDME	Prospective, multicenter, Phase 3	lucentis + sham laser	7 IVI	6.1	116
					Lucentis + laser	6.8 IVI; 1.7 laser	5.9	118
					Sham lucentis + laser	2.1 laser	0.8	111

[55]	READ-2	6 months	CMT OCT ≥ 250 *μ*m	Prospective, multicenter, Phase 2	Lucentis alone	4	7.2	42
			dDME and fDME		Laser alone	1.8	−0.4	42
					Lucentis + laser	2 IVI; 2 laser	3.8	42

[56]	READ-2	24 months	CMT OCT ≥ 250 *μ*m		Lucentis alone	9.3	7.7	33
		Above study [55] + 18 months	dDME and fDME	Prospective, multicenter, Phase 2	Laser alone; delayed lucentis	4.4 IVI; 1.8 laser	5.1	34
		+18 months			Lucentis + laser	4.9 IVI; 2 laser	6.8	34

[57]	RESOLVE	12 months	CMT OCT ≥ 300 *μ*m	Prospective, multicenter, phase 2	Lucentis	10.2	10.3	102
					Sham (no medication injected)	8.9 (sham treatments)	−1.4	49
					Note: rescue laser for both groups			

[58]	DA-VINCI	12 months	CMT OCT ≥ 250 *μ*m	Prospective, multicenter, Phase 2	Eylea (all arms combined)	9.3 IVI; 0.7 laser	9.7 to 13.1	175
					Laser alone	2.5	−1.3	44
					Note: rescue laser after month 6			

[59]	DRCR Protocol I: lucentis versus prompt or deferred laser	36 months	ciDME	Prospective, multicenter	0.5 mg lucentis + prompt laser	12 IVI; ≥ 1 laser, 100%	6.8	144
					0.5 mg lucentis + deferred laser	15 IVI; ≥ 1 laser, 46%	9.7	147

[40]	BOLT	24 months	CMT OCT ≥ 270 *μ*m persistent ciDME	Prospective, single center	Avastin alone	13 IVI	8.6	37
					Laser alone	4 laser	−0.5	28

[41]	PACORS	24 months	dDME	Retrospective, multicenter	Avastin alone	5.8	11.8	141
					Laser alone	2.2	4.8	120
					Avastin + laser	6.2 IVI*; 1 laser	8.2	157

*IVI: Intravitreal injection.
